# Drivers of cardiovascular disease risk factors in slums in Kampala, Uganda: a qualitative study

**DOI:** 10.1080/16549716.2022.2159126

**Published:** 2023-01-06

**Authors:** Rawlance Ndejjo, Paineto Masengere, Douglas Bulafu, Lydia Nabawanuka Namakula, Rhoda K. Wanyenze, David Musoke, Geofrey Musinguzi

**Affiliations:** Department of Disease Control and Environmental Health, School of Public Health, College of Health Sciences, Makerere University, Kampala, Uganda

**Keywords:** Alcohol, diet, physical activity, smoking, slums

## Abstract

**Background:**

Cardiovascular disease (CVD) risk factors are increasing in many sub-Saharan African countries and disproportionately affecting communities in urban slums. Despite this, the contextual factors that influence CVD risk among slum communities have not been fully documented to guide interventions to prevent and control the disease.

**Objective:**

This study explored the drivers of CVD risk factors in slums in Kampala, Uganda.

**Methods:**

This qualitative study employed focus group discussions (FGDs) to collect data among slum residents. A total of 10 FGDs separate for gender and age group were held in community public places. Discussions were audio-recorded, transcribed, and transcripts analysed thematically with the aid of Atlas ti 7.0. Study themes and sub-themes are presented supported by participant quotations.

**Results:**

Five themes highlighted the drivers of CVD risk factors in slum communities. (1) Poverty: a critical underlying factor which impacted access and choice of food, work, and housing. (2) Poverty-induced stress: a key intermediate factor that led to precarious living with smoking and alcohol use as coping measures. (3) The social environment which included socialisation through drinking and smoking, and family and peers modelling behaviours. (4) The physical environment such as the high availability of affordable alcohol and access to amenities for physical activity and healthy foods. (5) Knowledge and information about CVD risk factors which included understanding of a healthy diet and the dangers of smoking and alcohol consumption.

**Conclusion:**

To address CVD risk in slums, broad-ranging multisectoral interventions are required, including economic empowerment of the slum population, stress reduction and coping interventions, and alcohol legislation. Also, there is a need for community CVD sensitisation and screening as well as increasing access to physical activity amenities and healthy foods within slums.

## Introduction

Cardiovascular disease (CVD) risk factors are increasing in many sub-Saharan African countries, disproportionately affecting urban communities. Studies in East, West, and Southern Africa have found high odds of CVD risk factors, including obesity, hypertension, and diabetes [1–3]. The prevalence of CVD behavioural risk factors, including smoking, harmful alcohol consumption, inadequate physical activity, and inadequate fruit/vegetable consumption, is higher in urban settings [1]. In Uganda, although the estimated overall prevalence of hypertension in 2014 was 26.4%, the burden was higher in urban (28.9%) compared to rural areas (25.8%) [4]. Urban residents have also been shown to experience lower levels of physical activity and poor diets compared to their counterparts in rural settings [5,6]. For example, consumption of more than five servings of fruits and vegetables was reported at 13.8% in rural compared to 9.3% in urban areas [5]. The prevalence of alcohol consumption and smoking in urban Uganda is also high [7,8]. Within urban areas, CVD risk factors disproportionately affect inhabitants of informal settlements amidst high poverty levels, poor living conditions, and low access to social services [9,10].

Uganda is fast urbanising, leading to an increase in the number of urban residents and slum populations. Slums are characterised by several unfavourable conditions such as poor housing and environmental conditions, easy access to cheaper fast foods, and high levels of smoking and alcohol use amidst high poverty levels. These conditions have the potential to lead to an increase in non-communicable diseases (NCDs), hence requiring urgent interventions [10]. CVD risk factors common among slum residents elsewhere include high fat intake and low fruit and vegetable consumption [11,12], low physical activity [13,14], high tobacco smoking [15–17] and alcohol consumption [3], and increased levels of psychological stressors [18]. The contextual factors that influence CVD risk factors among slum communities are important to guide broad and specific interventions to prevent and control this growing public health concern. However, there is a paucity of in-depth studies to understand the context of Ugandan slums and how they may influence the burden of CVD. This study explored the drivers of CVD risk factors in slums in Kampala, Uganda.

## Methods

### Study design and area

This study utilised a descriptive qualitative design to explore the drivers of CVD risk factors in slums in Uganda using focus group discussions (FGDs) among community members. We used the constructivism paradigm [19] to understand how the interaction between individuals and their environment could influence CVD risk factors. The study was conducted in five slums in Uganda’s capital, Kampala. The city has a population of 1.7 million people, constituting about 25% of the country’s total urban population [20]. Kampala has five divisions of Kawempe, Rubaga, Nakawa, Central, and Makindye, and a slum community was selected from each, mostly those with the highest population sizes. The selected slums were Katanga in Kawempe, Kasubi in Rubaga, Namuwongo in Nakawa, Kisenyi in Central, and Nsambya-Gogonya in Makindye divisions.

### Data collection

Ten FGDs were conducted, two in each selected slum (one for each gender), among community members using a guide developed and informed by the broad themes of inquiry based on previous literature and the socio-ecological model. The socio-ecological model takes into consideration the individual (knowledge, attitudes, and skills), interpersonal (families, friends, and social networks), organisational (organisations, social institutions), community (relationships between organisations), and public policy (national, state, local laws, and regulations) factors that influence health behaviour [21,22]. We examined the influence of these factors on CVD practices and how they interact with each other to influence individual lifestyle choices. The guide, which was pretested in another Kampala slum not included in this study, had questions on CVD behavioural risk factors, including unhealthy diets, physical inactivity, smoking, and alcohol use, with probes to understand their drivers as well as community perceptions. We also collected socio-demographic information of the study participants, including age, education level, occupation, and duration living in the community. The FGDs were separated by gender (men and women) with seven among those aged below 40 years and three among those older than 40 years to facilitate discussion among demographically similar groups. FGD participants were approached physically and purposively selected by community leaders and mobilisers. The discussions were held in a public place with privacy within the community while adhering to COVID-19 control protocols, including mask-wearing and handwashing. The FGDs were led by two research team members (including RN, PM, DB – male – and LNN – female) with one mostly moderating the discussion and the other taking notes. The discussions lasted an average of 1 h and 20 min and were held in *Luganda*, the most widely spoken language in Central Uganda. The research team members were public health graduates with experience in conducting qualitative interviews. No relationship existed between the research team members and study participants. All FGDs were audio-recorded with participants’ consent. By the eighth FGD, no new information was obtained from the FGDs. However, all 10 planned FGDs were conducted to cover all the selected slums and increase representation of participant categories.

### Data management and analysis

The recorded interviews were transcribed verbatim and simultaneously translated into English. The transcripts were checked and verified by another team member while listening to some of the original audio recordings. Four study team members (RN, MP, BD, and LNN) read a few selected transcripts several times and generated a codebook which was discussed and harmonised to support the coding process. All interview transcripts were imported into Atlas ti version 7.0 software (GmBH, Berlin), and coding was done by the researchers following the semantic approach, with new codes appropriately added. At the end of the coding process, and guided by thematic analysis [23], similar codes were grouped into sub-themes which were synthesised into themes. Study themes are presented supported by participant quotations. Reporting for this study was guided by the Consolidated Criteria for Reporting Qualitative Research guidelines [24].

## Results

### Participant characteristics

We conducted 10 FGDs that involved 102 participants, 50 of whom were women. Among the participants, 45 were aged between 18 and 30 years, 59 had attained primary education as highest qualification, 50 were married, and 33 engaged in business (Table 1).

**Table 1.** Characteristics of participants.

Variable	Participants (*n* = 102)
**Slum of residence**	
Kasubi	22
Katanga	19
Kisenyi	19
Namuwongo	20
Nsambya Gogonya	22
**Gender**	
Men	52
Women	50
**Age category (years)**	
18–30	45
31–40	30
41–73	27
**Education level**	
None	5
Primary	59
Secondary	38
**Marital status**	
Never married	35
Married	50
Separated/widowed	17
**Religious affiliation**	
Christians	76
Muslim	22
None	4
**Occupation**	
Business	33
Casual labourer	15
Housewife	9
Unemployed	24
Others (farmer, hairdresser, driver, electrician, motorcycle rider, vendor)	21

### Drivers of CVD risk

Five themes described the drivers of CVD risk in slums. These are: poverty: a critical underlying factor; poverty-induced stress: a key intermediate factor; the social environment: amplifying risk factors; the physical environment: shaping choices; knowledge and information about risk factors: influencing action (Table 2). These themes and subthemes are described below.

**Table 2.** Summary of themes and sub-themes.

Themes	Sub-themes
Poverty: a critical underlying factor	•Eating to live
	• Walking and working to survive
	•Idleness – a facilitator of alcohol use and smoking
	• Smoking as a ‘blanket’ and alcohol a good company where there is no housing
Poverty-induced stress: a key intermediate factor	•Precarious and uncertain living leading to stress
	• Alcohol and smoking as stress coping measures
	• Crime and insecurity as a stressor and inhibitor
	• COVID-19 worsened poverty and increased stress
The social environment: amplifying risk factors	• Alcohol consumption and smoking for socialisation
	• Family and peers modelling behaviours
	• Influence of the significant other on behaviour
The physical environment: shaping choices	• High availability of affordable alcohol
	• Access to amenities for physical activity
	• Access to healthy foods
Knowledge and information about risk factors: influencing action	• Meats and a balanced diet constitute healthy eating
	• Does physical activity have health benefits?
	• The more you drink, the more you enjoy
	• Smoking: more harmful to the passive smoker
	• Health workers bridging the knowledge gap and influencing behaviour

### Poverty: a critical underlying factor

Four subthemes revealed how poverty was a critical underlying CVD risk factor. These were eating to live, walking and working to survive, idleness – a facilitator of alcohol use and smoking, smoking as a ‘blanket’ and alcohol a good company where there is no housing.

#### Eating to live

Almost all study participants agreed that poverty was a key underlying factor that affected the lifestyles they adopted some of which impacted their CVD risk. Participants mentioned that their incomes were low, and they lacked opportunities to economically empower themselves. Slum-residents decried the cost of living including housing that they deemed expensive as well as other needs including food. They mentioned that this kept them in a precarious poverty situation, leading to high levels of stress. Due to poverty, participants mentioned that food insecurity was an enormous threat to their health and well-being and that of their families. With challenges in access to food, most participants mentioned that they ate what they could access, and a healthy diet was not the foremost of their concerns. Some members avoided physical activity because it would make them eat more which food they did not have.

Here we live hand to mouth and can’t even afford to have all the meals in a day. So, when you get something to eat, regardless of the knowledge you have, you won’t care whether it is a balanced diet or has a lot of fats … . We simply just eat. (FGD 8, man, 31 years)

#### Walking and working to survive

Participants expressed that because they could not afford transportation means, including commercial motorcycles, they usually walked long distances. This, together with engaging in manual work such as working at sites, carpentry, and selling and hawking merchandise, especially among men, and house chores among women ensured that they were sufficiently active. There were a few reports of leisure-time physical activity consisting of visiting gyms mostly by young men, but low incomes usually made access harder and irregular.

The hill really helps us do physical exercise because we all have to go to the market, and we cannot afford paying for a commercial motorcycle. Whatever route from this slum to the market is hilly. We go uphill, reach some areas, and rest, and then continue uphill. During the return journey, we come walking fast down the slope. (FGD 1, woman, 58 years)

#### Idleness – a facilitator of alcohol use and smoking

Study participants noted that not having jobs and other activities to engage in made them idle with free time on their hands to drink and smoke which they would not have done if the situation was different. They noted that the lack of jobs or income pushed them to gather at communal places to pass time.

We are idle 24 hours a day, 7 days a week and that is why we smoke. I am a smart and knowledgeable person; if I had a job, I would not touch any [alcohol] bottle because I would return from work tired and just take a shower and sleep. But being idle with people who just keep buying you alcohol is a different story. So, if we had jobs, we would be too busy to do that. (FGD 7, man, 30 years)

#### Smoking as a ‘blanket’ and alcohol a good company where there is no housing

Due to irregular income, community members sometimes found themselves with nowhere to spend the night, especially in slums where they required daily payments to access public dormitories. This pushed them to spend late evenings at bars drinking alcohol. They also smoked cigarettes and marijuana which they termed as ‘a blanket’ to ‘shield them from the night’s cold and to not feel the mosquito bites. As the environment was usually risky, especially for the women, they sometimes engaged in transactional sex to obtain money and/or access the housing facilities.

Some people have no homes and sleep outside; they have to work during the day, but sleep in the bar at night. Without a place to sleep; they drink a few bottles, dose off from the bar and spend the night partying from one bar to the other. They will then go hustle more (work tirelessly) the following day and return. (FGD 7, man, 29 years)

### Poverty-induced stress: a key intermediate factor

Poverty-induced stress was a key intermediate CVD risk factor in the slums. This was explained by four subthemes: Precarious and uncertain living leading to stress, alcohol and smoking as stress coping measures, crime and insecurity as a stressor and inhibitor, and COVID-19 worsened poverty and increased stress.

#### Precarious and uncertain living leading to stress

Study participants reported that stress was a major issue within the slums attributed to a multitude of factors ranging from high poverty levels, poor living conditions, inadequate opportunities, low income, lack of necessities, high indebtedness, and relationship challenges. This led to their dissatisfaction with life, and they wondered whether they would ever get out of such a trap. The inability of men to provide for their families was also a source of stress and sometimes led to them neglecting their responsibility at home or violence. Among women, their inability to provide for their children caused them a lot of worries, anxiety, and stress as sometimes they were the sole breadwinner when their husbands lacked opportunities or abandoned the family. Participants mentioned that slums were characterised by many female-headed households, a high number of children, and young mothers/parents which they attributed to their circumstances. Poverty and stress also impacted marriages and relationships with the resultant instability compounding stress among both men and women. With a sense of abandonment, some participants wondered if living healthy was necessary at all.

Some [youths] have had children early and have many responsibilities that they cannot manage. We have friends who have had a mental breakdown due to this pressure. (FGD 7, man, 36 years)

#### Alcohol and smoking as stress-coping measures

As a means to cope with the high levels of stress they endured, participants resorted to consuming alcohol and drugs frequently in far higher quantities. They said that alcohol and drugs helped them momentarily forget their problems, including reducing appetite for food, especially when they felt that no one cared to offer them a helping hand. Alcohol consumption was also a precursor for use of drugs and vice versa, and these pushed some community members to engage in risky behaviours, including transactional sex and crime. A few participants reported other coping mechanisms, including engaging in sports and music which increased their physical activity, prayer, or unburdening with friends, and this kept them away from unhealthy coping activities.

Someone would want to drink some [alcohol] to be able to forget that which is pressurising them and it is hard to find a youth here who does not use alcohol. They are just a handful. They know they can get a portion of waragi (local spirit) at 500 shillings (15 US cents) to drink to the point of forgetting their hunger. You know when hunger strikes you and you’re conscious, you can even eat up yourself [group laughs], but when you have taken some alcohol, you can forget about it. You are like someone satisfied; you will pass time as dusk approaches. That I think is the biggest reason people here drink. (FGD 7, man, 36 years)

#### Crime and insecurity as a stressor and inhibitor

The slum community had a high rate of crime and violence due to the high population density, joblessness, and poverty. Participants said that crime was high which often led to law enforcement in the community. This sometimes meant that some slum residents, especially young men, were arrested and imprisoned. This finding, the women said, was another cause of stress as their husbands and children were arrested often, and they required money to bail them out of prison. Also, due to crime and violence, some participants noted that it was risky and unsafe to engage in physical activity within the community including jogging.

We mostly do physical exercise during the day and will not get out at night to go running, otherwise you will run into gangs who will beat you up. Most youths here do not work, they become active at the crack of dawn. (FGD 10, man, 21 years)

#### COVID-19 worsened stress and affected lifestyles

The COVID-19 restrictions impacted slum residents’ lifestyles, reducing incomes, and increasing stress and uncertainty. The restrictions that were implemented to curb the pandemic negatively impacted employment and incomes as many lived hand to mouth, affected diets, and increased reliance on alcohol and drugs to cope. The restrictions on gatherings also negatively impacted group physical activities, and many community members could no longer partake in such activities. Participants who also used to meet their physical activity levels through travel and work-related routines were no longer able to do so due to reduced avenues such as work. On the other hand, some participants reported an increased activity such as more walking time due to the inability to access transportation owing to lockdown measures.

I am a teacher and for the entire 2 years of the COVID-19 lockdown, I have been home sitting with no opportunity to exercise. I spend the day sleeping indoors, I get out to eat and then get back inside. I used to do my exercises by walking to my workplace daily and back, but ever since the lockdown was instituted, I stopped exercising. There is nowhere to exercise from. (FGD 5, woman, 50 years)

### The social environment: amplifying risk factors

Three interpersonal factors played a part in CVD risk within the slums. These were alcohol consumption and smoking for socialisation; family and peers modelling behaviours; and influence of the significant other.

#### Alcohol consumption and smoking for socialisation

Within the communities, alcohol consumption was considered the greatest tool for socialisation, and many slum residents spent time in the bars catching up with what was happening in the community while drowning in their stress. It was noted that community members found it easier to buy each other alcohol or share that which was available making it harder to monitor consumption levels. Several participants emphasised that even without money, it was quite easy for community members to buy each other alcohol with drinking starting earlier than in many areas. Participants mentioned that indulging in drinking alcohol was also a distraction when they could not obtain food. Relatedly, especially among youths and men, drugs were an additional avenue for socialisation sometimes done concurrently while smoking in the case of cigarettes or in secluded places for prohibited drugs like marijuana. Indeed, male youths used alcohol much more than other community groups and reported more physical activity mostly influenced by their social environment.

Our community has an increased number of alcohol users, especially the youths. You find a youth aged 20 years old with a bottle of waragi (spirit) in their back pocket instead of a wallet. You can live the whole day after taking waragi of five hundred shillings, but that is not good for your health. (FGD 10, man, 21 years)

#### Family and peers modelling behaviours

Peers and social networks within slums greatly influenced lifestyle practices, including physical activity, alcohol consumption, and smoking. For example, sometimes youth came together to exercise as a group or constituted sports teams which kept them physically active. In some households, the family positively influenced the foods prepared in the home, especially those with hypertensives or diabetics. Other families also engaged in physical activities together.

I do physical exercise with my children. We play dodge ball and skip the rope. Sometimes they swing the rope as I jump. (FGD 5, woman, 27 years)

Other community members noted that a low involvement of parents in their children’s lives or their negative influence, harsh slum conditions, or peers drove children and youths to adopt undesirable lifestyles, especially unhealthy diets, use of alcohol, and drugs. Some participants also reportedly abstained from smoking cigarettes or other drugs, attributing it to the influence of their family while growing up which had shaped their choices.

#### Influence of the significant other on behaviour

In some women FGD groups, it emerged that their physical activity choices were influenced by their husbands who they said preferred them fatter. For this reason, some avoided physical activities as they did not want to lose weight.

My husband wants me to grow fat, so when I start doing exercises, I may lose weight and that’s the only reason I am not exercising. He says a fat woman looks good for a home, appears to be complete and the man can be proud to have them. So even at home, I do not engage in physical exercise. (FGD 5, woman, 27 years)

### The physical environment: shaping the choices

Three physical environment factors influenced the CVD risk in the slums. These were high availability of affordable alcohol; access to amenities for physical activity; and access to healthy foods.

#### High availability of affordable alcohol

Study participants mentioned that their communities had a high density of alcohol outlets, most of which sold it in small cheaper quantities. This increased availability, access, and consumption of alcohol. Some community members also mentioned that they found it cheaper to buy alcohol than food as they dealt with stress and only thought about food afterwards.

Waragi (local spirit) is only 500 shillings (15 US cents). You will not find anyone here buying a bottle of beer but cheaper spirits such as Kyarenga, Twebereremu, Sanyuka [local names] of between 500 to 1000 shillings. No one is going to buy four bottles of beer here but many locally brewed options are cheaper. (FGD 6, man, 28 years)

#### Access to amenities for physical activity

Study participants generally agreed that the slum environment characterised by congested housing, open drainages, and waste did not present a suitable setting for outdoor physical activity. The houses were also too small without a compound which reduced suitability for indoor physical activities. Those who had bigger houses mentioned that they found it easier to do indoor activities.

My house is big enough, and I also have relatively small furniture. The children sleep in their own room, so I have space to do physical exercises from the sitting room. (FGD 5, woman, 33 years)

In some slums, they had access to playgrounds and gyms which facilitated engagement in physical activity. However, even with playgrounds, sometimes access to balls was deemed hard, especially among women who also said that gym activities such as boxing and equipment mostly favoured men. Paying a fee to use the gym also increased difficulties in access.

#### Access to healthy foods

Although some slums were near the market which facilitated access to healthier food, in others, access to fruits and vegetables was harder compared to most fast-food options which were readily available and cheaper. Fruits and vegetables were also said to be seasonal and thus their prices fluctuated based on availability. This too influenced healthy eating for CVD prevention.

I can take a full week without eating fruits because I cannot afford to spend five hundred shillings to buy a small piece of jackfruit yet the children are waiting for food at home. (FGD 5, woman, 32 years)

##### Knowledge and information about risk factors: influencing action

Among study participants, five sub-themes described how knowledge and perceptions of behaviours influenced CVD risk. These subthemes were meats and a balanced diet constitute healthy eating; Does physical activity have health benefits? The more you drink, the more you enjoy; smoking is more harmful to the passive smoker, and health workers bridging the knowledge gap and influencing behaviour.

#### Meats and a balanced diet constitute healthy eating

Participants expressed that they did not understand what constituted a healthy diet with most valuing eating meats and others stating that they simply needed to eat a variety of foods to ‘balance their diet’.

Healthy feeding requires that you eat a variety of foods. Fine, the situation might have been hard for you on the day and may only afford chapati and beans of one thousand shillings, but you don’t have to eat it again. You should change and eat something else. That can keep the body healthy. But whenever you eat one kind of food, the body won’t function well. (FGD 8, man, 37 years)

#### Does physical activity have health benefits?

Slum residents also did not have satisfactory knowledge about the healthy levels and benefits of physical activity. As a result, they reported not doing physical activity. It was also evident that some slum residents did not consider work-related activities as physical exercise.

Physical exercise is rare in the community because we lack knowledge. Only a few have understood that physical exercise has some health benefits. I learnt the little I know from a health worker that is why I say that we lack knowledge. (FGD 2, woman, 32 years)

Among those that appreciated the importance of physical activity and reported having more time, they were more likely to report engaging in physical activity. Moreover, the incorporation of physical activity within daily routines such as doing so very early in the morning or late in the evening was a key facilitator of engaging in physical activity. The media also played a key role in facilitating routine physical activity.

As for me, I do exercises in my house following the guide session on the television at 5:00 am every day. (FGD 5, female, 33 years)

#### The more you drink, the more you enjoy

Slum residents did not know about the acceptable levels of alcohol consumption as they emphasised that it was immeasurable. They also associated concentrated alcohol such as spirit to have the potential to burn fats and reduce weight which sometimes led to harmful drinking.

You cannot measure it. The more you drink, the more you enjoy until you find yourself falling off. I do not know how you would measure alcohol intake levels … . I once fell ill without knowing the real diagnosis, upon screening, they said that my blood pressure was raised, and some people advised me to use some gin made from bananas. ‘Real concentrated gin will reduce fats’ they said. (FGD 2, woman, 52 years)

#### Smoking is more harmful to the passive smoker

Some participants also reported misconceptions about the harms of smoking and some could not relate it to heart disease. Some also mentioned that even when they smoked, it was more harmful to those around them than themselves.

#### Health workers bridging the knowledge gap and influencing behaviour

The interaction of slum residents with health workers contributed to a better understanding of CVD and adoption of healthy lifestyles, especially when faced with some risk factors. Participants who reported having a CVD risk factor such as hypertension and/or diabetes noted that through their interaction with the health facilities, they had appreciated the importance of healthy lifestyles. They generally exhibited better knowledge of CVD and its risk factors, usually describing the need to reduce salt and sugar intake and increase consumption of vegetables as well as the need to increase physical activity levels and reduce alcohol consumption and smoking. Participants’ health status also influenced what forms of physical activity they engaged in.

I eat fruits often because I was told by the health worker to eat lots of them and vegetables more than the other foods. (FGD 5, woman, 73 years)

Those things that we take have no value and only leave you with diseases in that they take you to the hospital and you are asked, what caused the disease you have?, Do you smoke cigarettes, khat?. And they inform you that the disease is from all those things. They treat and caution you not to ever use them again. (FGD 9, man, 44 years)

However, the community noted that their health-seeking behaviours were poor. There was also generally a less focus on NCD risk factors due to the higher prevalence of communicable diseases in the area that also influenced community programmes.

**Figure 1.**
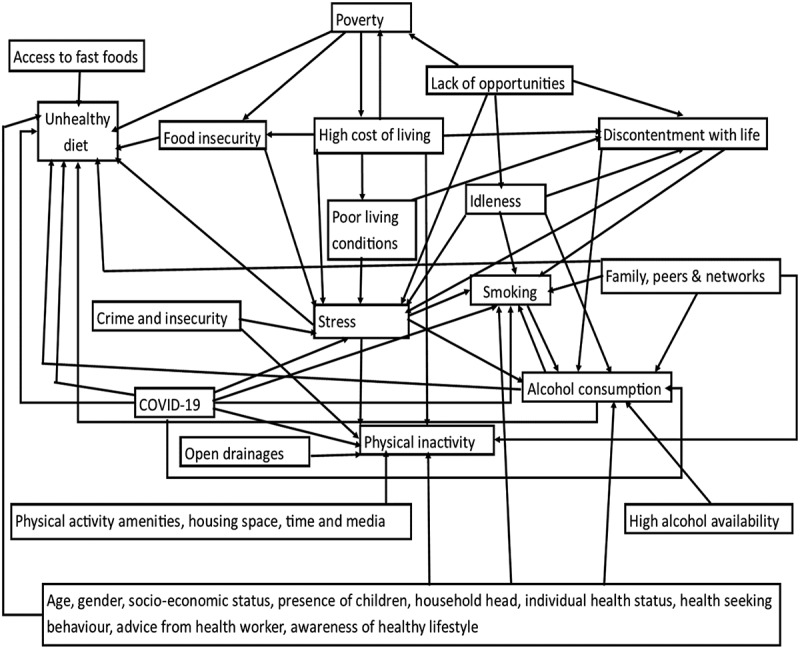
Drivers of CVD risk in slums and their interconnectedness.

Figure 1 summarises the drivers of CVD risk and their interconnectedness within the slum communities informed by the data.

## Discussion

This study explored the drivers of CVD risk factors in slums in Kampala, Uganda. We found that poverty was the key underlying factor that impacted lifestyle practices, including diet. Poverty also induced stress which contributed to alcohol consumption and smoking habits as coping strategies. Moreover, the social environment also played a key role, especially in reinforcing CVD-related habits and behaviours. The other key drivers of lifestyle practices were the physical environment within the slums, and knowledge and information about CVD and risk factors. Broad-based interventions tackling these multiple social determinants of health on which unhealthy lifestyle practices thrive are required to deal with CVD in slum communities.

The study findings represent a vicious cycle of highly prevalent CVD risky lifestyles stemming from poverty as a critical root cause. Slum communities are characterised by socio-economic inequalities with residents of low income, low education levels, unemployed, and from unstable families usually living in squalid conditions [25]. These circumstances translate into high poverty levels which increases the vulnerability of slum communities [26]. Consequently, through a sequence of events and other intermediate factors, risky CVD lifestyles arise and/or are maintained. In the study community, high poverty levels led to stress, a key risk factor for CVD. Chronic stress can bring about metabolic dysfunction, leading to high blood pressure and atherosclerosis which can eventually translate into CVD [27]. Due to limited access to comprehensive quality care, CVD and related complications lead to high out-of-pocket expenditure crippling household finances and further perpetuating the poverty cycle. To cope with stress, community members resorted to excessive alcohol intake and smoking. Alcohol use and smoking are highly prevalent among adults in urban slums in Uganda and elsewhere [28–30]. Other contributors to the high alcohol and smoking habits in the slums were idleness due to unemployment, lack of housing, and inadequate knowledge of the dangers of these habits. The role of interpersonal factors, including social networks and peers, was also key in forming, normalising, and sustaining alcohol drinking and smoking as great socialisation tools. Indeed, male youths were more likely to consume alcohol and smoke and were more physically active compared to their counterparts. In dealing with broader CVD risk factors within slum communities, alcohol and smoking reduction interventions should target young males while physical activity strategies are required for females and older community members. Overall, reversing the observed factors requires economic initiatives to empower slum communities and support them in alleviating poverty and its induced stress. Stress reduction interventions are also required to support communities to cope better, and these should be widely encompassing to influence their peers and social networks. Overall, multisectoral interventions bringing together various sectors, including urban planning, labour and social development, gender, health, and environment, among others, are critical to comprehensively address CVD risk drivers in slums.

The physical environment, including the high availability of affordable alcohol, access to physical activity amenities, and access to healthy foods, also influenced CVD risk. Interventions that regulate alcohol manufacture, cost, packaging, and sale including age of access could go a long way in reducing community exposure. In 2017, the Government of Uganda banned the sale of alcohol in sachets and 200-millilitre packages which although led to reduced alcohol availability [31], the practice has persisted. The National Alcohol Control Policy was later introduced in 2019 to prevent and reduce alcohol harm but gaps still exist in operationalising it. Plans are currently underway to introduce an Alcohol Control Act to regulate the alcohol industry which may further contribute to reduced alcohol availability and consumption if well enforced. Also, increasing access to physical activity amenities and healthy foods can support the adoption of healthy behaviours within slums and can be a focus of interventions. There were some positive health behaviours reported, including walking and working and consumption of vegetables. However, these activities were mostly non-deliberate and were influenced by circumstances of low income. Increasing awareness of the importance of such behaviours could improve the appreciation of these behaviours in health promotion and influence their adoption.

We also found that knowledge and awareness of CVD and risk factors were low among slum residents with poor awareness of risks and benefits of healthy lifestyle practices. Although awareness of possession of CVD risk was reported as a facilitator for healthier lifestyle choices, this awareness is low in many Ugandan communities [32,33]. With challenges of access to healthcare in slums, awareness is likely lower which requires urgent attention. Previous research has shown knowledge gaps on CVD and risk factors among slum residents, including in Kenya [34] and India [35]. Interventions to deal with CVD risks within the community should include sensitisation and awareness-raising to provide community members with accurate information regarding CVD and the importance of healthy lifestyle practices. Other approaches include community screening for CVD risk factors and the use of mHealth-assisted community-based CVD risk screening techniques as has been the case in Kenya [36,37], India [37], and Bangladesh [38]. Moreover, in the community, health workers were highlighted as a critical source of CVD information. However, since health workers usually target a few community members who visit health facilities, community resources, including community health workers, local and religious leaders, among others, can be trained and supported to conduct health promotion for CVD activities. Such community-based efforts can lead to a reduction in CVD risk [39,40]. The media is another avenue that was reported as a source of information and can support health education for CVD prevention. As knowledge alone is not sufficient to lead to practice, greater efforts are required to create opportunities and enabling environments to facilitate healthy lifestyle practices. Multisectoral approaches that bring together governmental, non-governmental, and the private sector players across various disciplines and involve the community are more likely to be successful in addressing these slum health CVD determinants.

This study was informed by the socio-ecological model to support exploring a breadth of drivers of CVD risk. The FGDs were separate for men and women within closer age ranges that supported their self-expression. As this study was only conducted in slums in Kampala, the transferability of findings may be limited though the contextual issues highlighted are similar to those in other slums in the country. The study was also conducted 2 months after the partial lifting of the second lockdown restrictions which could have influenced participant experiences. However, we focused on broader community experiences beyond the COVID-19 impact. Overall, the study findings offer great insights to inform CVD prevention interventions in slum settings.

## Conclusions

The key drivers of CVD risk factors in slums were poverty, poverty-induced stress, interpersonal factors, physical environment, and knowledge and information about risk factors. Among these, poverty was the critical underlying factor that greatly influenced the other risks. Broad-ranging multisectoral interventions, including economic empowerment of the slum population, stress reduction and coping interventions, and alcohol legislation, are required to prevent CVD in slums. In addition, there is a need for community CVD sensitisation and screening as well as increasing access to physical activity amenities and healthy foods within slums.

## Data Availability

The data that support the findings of this study are available on request from the corresponding author [RN]. The data are not publicly available so as not to compromise the privacy of research participants.

## Paper context

Cardiovascular disease (CVD) risk factors are on the rise in many low-income contexts disproportionately impacting urban communities. However, gaps exist in understanding the drivers of CVD risk factors, especially in informal settlements to inform potential response interventions. Through focus group discussions among slum communities, we have uncovered the potential drivers of CVD risk factors and their interconnectedness and highlight the need for multisectoral strategies to effectively deal with them.
